# Method for UAV propeller characterization using frequency analysis of Lidar signals

**DOI:** 10.1007/s00340-025-08533-9

**Published:** 2025-08-01

**Authors:** Adrien P. Genoud, Topu Saha, Joseph Torsiello, Ian Gatley, Benjamin P. Thomas

**Affiliations:** 1 Department of Physics, New Jersey Institute of Technology, Newark, NJ 07102 USA; 2 Universite Claude Bernard Lyon 1, CNRS, Institut Lumière Matière, UMR5306, 69622 Villeurbanne, France; 3 Department of Physics, Temple University, Philadelphia, PA 19122 USA

## Abstract

The rapid proliferation of commercial unmanned aerial vehicles (UAVs) poses growing security, safety, and privacy challenges. This paper presents a novel frequency-domain analysis methodology to extract mechanical signatures of UAVs using backscattered optical signals from drone propellers. Through both simulations and experimental validation, the feasibility of retrieving key mechanical signatures, including the propeller's rotational speed (RPM) and the number of blades, was demonstrated. These signatures are a first step towards the real-time identification of drone models and provide insights into drone’s flight behavior. The methodology, tested here with small toy drones, offers promise for real-world deployment of drone monitoring systems, complementing traditional detection techniques by operating in various atmospheric conditions. Additionally, harmonic and frequency peak analysis may allow for future improvements in trajectory tracking and payload detection. This work opens new possibilities for integrating lidar-based UAV characterization into both civilian and military airspace security frameworks.

## Introduction

The widespread availability and the democratization of commercial unmanned vehicles (UAV) and small unmanned aerial systems (sUAS), publicly known as drones, have introduced significant concerns regarding safety, security and privacy over this last decade [1–3]. These issues are increasingly relevant, not only in civilian contexts but also in military and high-profile public events. The recent Paris 2024 Olympic Games have prompted a heavy deployment of counter-drone technologies to address potential threats. These technologies include radar systems, RF jammers, and counter-UAV lasers, all aimed at protecting public spaces from malicious drone activities. Similarly, recent developments in the Ukraine-Russia conflict underscore the critical role of drone detection and countermeasures for military personnel, as drones are employed for both offensive operations (e.g., transporting explosive devices), reconnaissance (e.g., equipping observation cameras) and electronic warfare (e.g. signal jamming and communication relays).

Due to the large spectrum of threats that UAVs can cause, the last decade has seen a race to develop instruments to detect, identify and potentially neutralize drones. While the defense industry has made considerable progress in developing advanced counter-drone technologies, academia has made fewer effort in this area. As a result, there is a gap between the cutting-edge technologies developed by the private sector and the current state of the art in academic research on UAV detection and mitigation. Different methods have been developed over the last decade, such as detecting and even tapping wireless communications using radio frequency sensors [4–7] detecting the sound of UAV propellers and motors [7, 8], visible and infrared cameras [9–14] or radars [15–20]. In particular, millimeter-wave radar systems have emerged as powerful tools for UAV detection and motion analysis, especially through the use of micro-Doppler signatures generated by rotating propellers [21–23]. These systems are capable of detecting subtle motion features and have demonstrated good robustness in complex environments, where techniques like time–frequency decomposition, deep learning, spectrograms and target classification via motion profiles can distinguish drones from birds or other moving objects [3, 23–27].

The increasing prevalence of UAVs in sensitive airspaces poses significant operational risks, particularly where traditional detection systems fall short in differentiating between commercial and potentially hostile drones. Radio frequency-based detection systems, for instance, are prone to interference and may not provide sufficient data to uniquely identify specific drone models. Visual systems, while useful for larger or slower-moving drones, are limited by weather conditions, the range of detection and their diminished effectiveness in low-light environments or during nighttime operations.

Lidar technology is a potential avenue to collect data on incoming drones [28–31]. Various lidar-based approaches have been explored in this context, including pulsed lidar, gated detection coherent Doppler techniques [32–34], which are particularly suited for distance-resolved detection and velocity measurements. In this work, a continuous-wave (CW) backscattering lidar system is employed to investigate a different aspect of UAV characterization, focusing on the analysis of the temporal modulation of the backscattered signal induced by the rotating propellers. Backscattering lidar can capture reflections from the target drone, enabling remote sensing of structural characteristics and motion signatures. Unlike traditional radar, which primarily detects movement or bulk shape, the interaction of infrared laser light with the propeller blades provides detailed information on the drone’s mechanical components.

The aim of this paper is to propose a new methodology contributing to the remote identification of commercial drones, particularly focusing on the analysis of backscattered light from drone propellers. By analyzing the signal periodicity induced by the rotating blades, the proposed methodology can yield real-time insights into propellers rotation speed and blade numbers. These mechanical signatures can serve as identifiers of specific drone models, making this technique highly valuable for real-time airspace monitoring and security applications. This methodology has been used with great success in the monitoring of flying insects using entomological lidars and many of the methodologies used in this field can be transposed to flying drones [35–39].

This contribution presents a methodology to characterize some of the properties of flying commercial UAVs and sUAS using an infrared optical system. By doing a frequency analysis of backscattered signals from flying drones, the rotation per minute of the propellers can be retrieved as well as the number of blades on the propeller. An experimental proof of concept of this methodology is provided using a short-range optical system in a laboratory setup on small commercial. This work provides a complementary approach to conventional detection systems, offering the potential for deployment in diverse environments, including urban settings and sensitive civilian infrastructure. The continuous-wave (CW) backscattering optical system presented in this study was specifically designed for controlled laboratory measurements, with the aim of validating this novel signal analysis methodology for UAV characterization. This experimental setup does not offer any range resolution, however the core principle of extracting propeller characteristics from the frequency content of the backscattered signal remains valid and applicable to range finding methodologies, such as phase-shift range finding lidar or frequency-modulated continuous wave lidar [40–43].

## Materials and methods

**Fig. 1:**
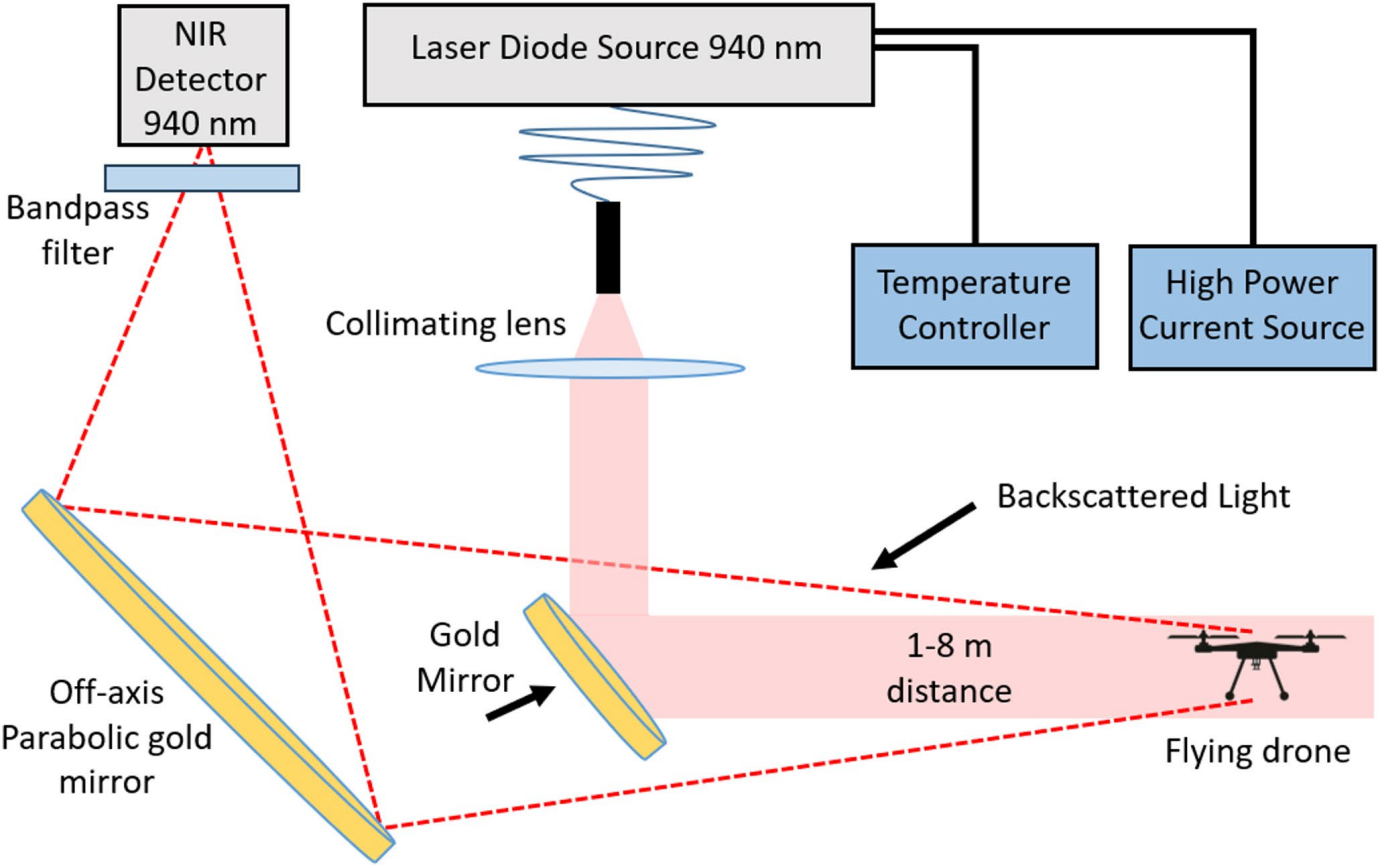
Optical layout of the lidar for the characterization of drones

Figure 1 presents the optical layout of the lidar system. A prototype was designed to make measurements at short range, between 1 and 8 m, in a laboratory setup. A continuous wave (CW) infrared laser diode source emitting at 940 nm wavelength and 5 W power is used. The infrared beam from the source is reflected toward the drone using a 7 cm diameter off-axis parabolic gold mirror. The light backscattered by the drone is collected and focused on an InGaAs amplified photodetector. The laser beam, parabolic gold mirror, and collecting optics are set to be coaxial. Signals are recorded using a 16 bit 250 MS/s 125 MHz bandwidth digitizer. It is important to note that the presented optical configuration was optimized for laboratory use, with a low laser beam divergence, designed to maximize signal-to-noise ratio in a short-distance setting. The drone's position relative to the beam was controlled to ensure consistent alignment through the laser path. While this setup allows for high-fidelity signal acquisition under well-defined conditions, it does not incorporate the necessary UAV detection and tracking capabilities that would be required in real conditions.

Equation 1 below presents the general expression for the backscattered intensity from a drone at a distance *d.*

1 $$I\left(t,d\right)= \frac{K}{{d}^{2}}*{I}_{0 }*{\sigma }_{tot}\left(t\right)+{I}_{B}$$

where ${\sigma }_{tot}$ is the total backscattering optical cross-section of the drone, function of the time $t$. $K$ is a constant taking into account the size of the off-axis parabolic mirror, collecting efficiency of the telescope, and optical transmission or reflection coefficients of the sending and collecting optics. ${I}_{0}$ is the initial power of the laser beam while ${I}_{B}$ is the background light intensity received by the detector.

This experimental setup was chosen to allow for the frequency content of the backscattered signal to be evaluated under controlled conditions. In principle, the proposed frequency-domain analysis does not require knowledge of the target distance, as long as the signal-to-noise ratio is sufficiently high to resolve the harmonic structure. However, in practical applications, range information may be valuable for spatial localization or signal calibration into optical cross sections. Distance measurements could be obtained by superimposing a commercial laser rangefinder along the optical axis of the CW laser [44, 45], or directly by modulating the CW source and retrieving the distance using the phase shift on the backscattered signal [46, 47].

The total optical cross-section of the drone can be decomposed into the sum of the propellers’ optical cross-section ${\sigma }_{p}$ and the optical cross-section ${\sigma }_{b}$ of either some parts or the whole drone’s body, as described in Eq. 2. In this case, it is assumed that the laser beam diameter is large enough to fully encompass the drone.

2 $${\sigma }_{tot}\left(t\right)={\sigma }_{p}\left(t\right)+{\sigma }_{b}$$

Due to the rotation of the propellers, the apparent optical cross-section of the blades within the laser beam's field of view will vary with time. Given that typical drone propeller’s RPM are in the range of thousands, the time-dependent variation of ${\sigma }_{p}$ occurs on a much faster timescale than that of ${\sigma }_{b}$. Consequently, while both ${\sigma }_{p}$ and ${\sigma }_{b}$ exhibit time dependence as the drone moves through the laser beam, only the rapid variations in ${\sigma }_{p}$ are of particular importance for our application. Specifically, the temporal fluctuations in ${\sigma }_{p}$ occur on the millisecond scale, whereas variations in ${\sigma }_{b}$ are on the order of the second, making the latter negligible for the purposes of this study.

**Fig. 2:**
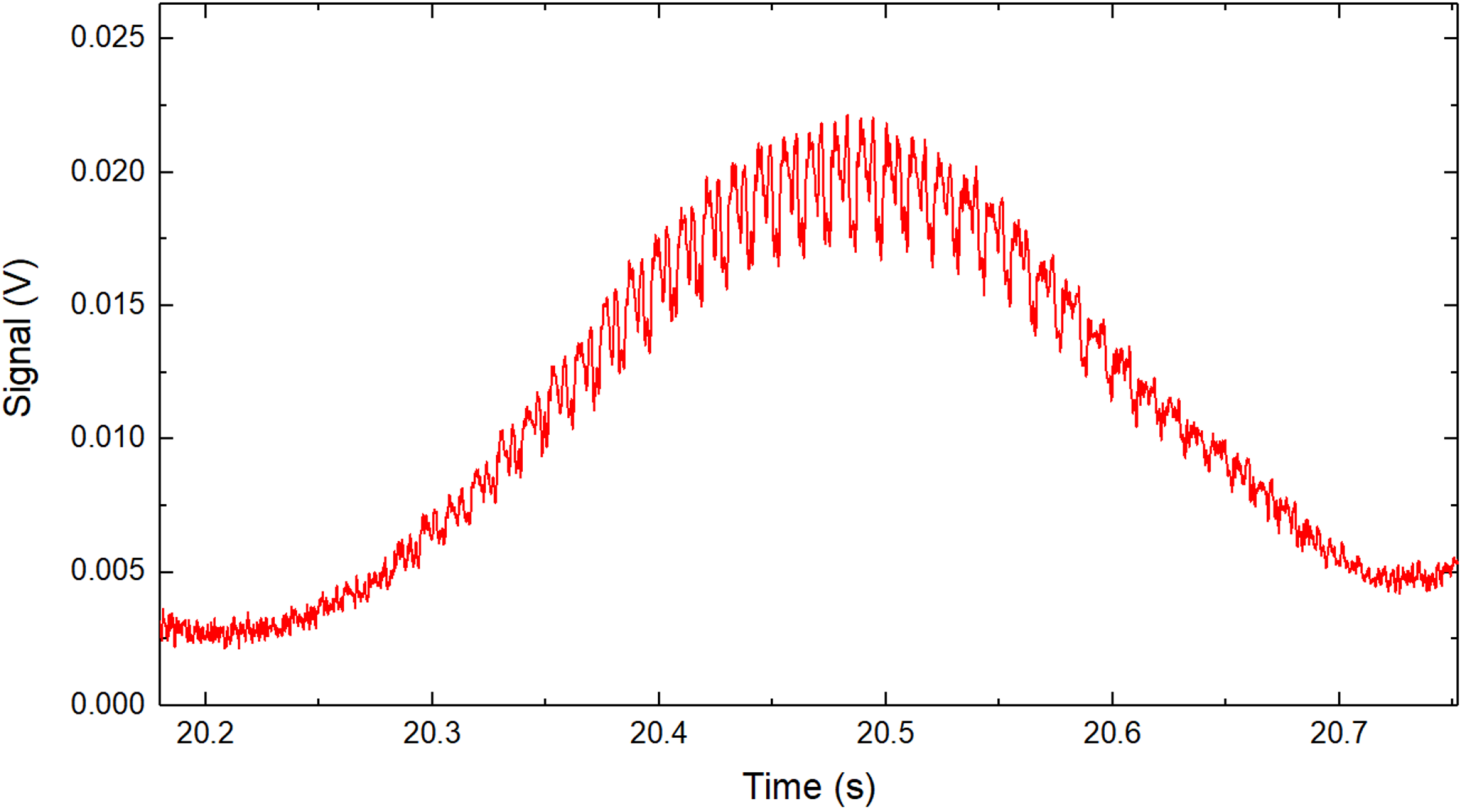
Raw backscattered signal of a Cheerson CX-10 RC drone transiting through the laser beam showing the amplitude modulation of the signal caused by the rapid movement of the propeller

Figure 2 presents a measured backscattered signal from a UAV (Cheerson CX-10 RC) transiting through the laser beam. Raw signals are characterized by their overall Gaussian shape as the drones were transiting through the laser beam, however longer signals could be obtained by either tracking the drone movement or having the drone hovering at one position. Oscillations of the signal amplitude are caused by the rotations of the propellers resulting in change in ${\sigma }_{p}$ over the time of transit.

In total 3 commercially available drones have been thoroughly studied, each having a total of four propellers:

- Hubsan Q4 Nano H111 Quadcopter, which has 2 blades per propeller
- Dwi Dowellin Mini Drone, which has 3 blades per propeller
- ELF Mini RCtown Drone, which has 4 blades per propeller

Analysis of the backscattered signal can be used to extract the following key features of the drone:

- Propeller Rotation Per Minute (RPM)
- Number of blades on the propeller (N_b_)

## Results and discussion

**Fig. 3:**
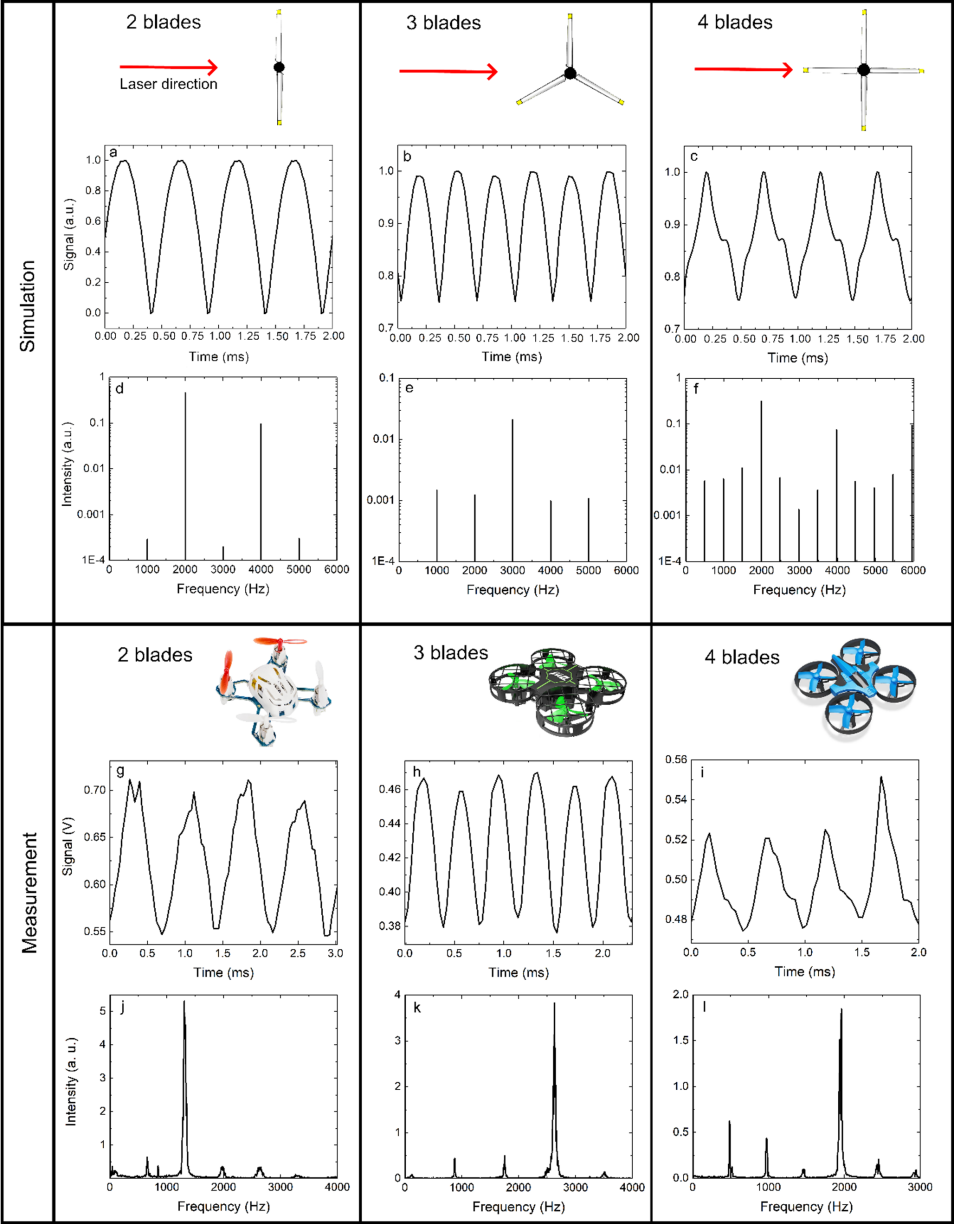
Simulated and experimental signals from drones with 2, 3 or 4 blades per propellers showing that the frequency analysis can be used to determine the RPM and number of blades for the drone’s propellers. Plots a, b and c display the simulated optical signal of the 2,3 and 4 blade-propeller, respectively. Plots d, e and f display the Fourier transform of the simulated optical signal of the 2,3 and 4 blade-propeller, respectively. Plots g, h and i display the measured optical signal of the 2,3 and 4 blade-propeller, respectively. Plots j, k and l display the Fourier transform of the measured optical signal of the 2,3 and 4 blade-propeller, respectively

By doing a frequency analysis of the backscattered signal from the drone, this methodology can retrieve the RPM and the number of propeller blade. A simulation based on 3D models of drone propellers was designed with varying numbers of blades (2, 3, and 4 blades) as shown in Fig. 3. The simulation calculates the apparent cross-section of the blades within the field of view of the incident laser beam, allowing us to model the backscattered optical signal originating from the propeller’s blades, i.e. the amplitude modulation described in Fig. 2. The relationship between the optical signal and the apparent cross-section is described by Eqs. (1) and (2), demonstrating that the signal is directly proportional to the time-varying apparent cross-section of the propeller.

In the simulation, the propellers operate at an arbitrarily fixed speed of 60,000 RPM, equivalent to 1000 rotations per second (Hz). The simulated time-domain data spans a thousand full propeller rotations, which is then analyzed in the frequency domain using a Fourier Transform. This analysis reveals both the fundamental frequency of the signal, corresponding to the drone's RPM, and key harmonic components that provide information about the number of blades.

Experimental measurements using the optical setup illustrated in Fig. 1 were also performed for comparison. These measurements were taken from the three aforementioned commercial drones equipped with propellers having either 2, 3, or 4 blades per propeller. In the case of the 3-blade drone, the protective cage surrounding the propeller was removed to ensure unobstructed laser access to the propellers.

The frequency analysis of both the simulated and measured signals provides critical insights. First, the fundamental frequency ${f}_{1}$ of the propeller, expressed in Hz (equivalent here to rotation per second), can be directly retrieved from the signal. This frequency is related to the RPM and the angular velocity $\Omega$ as expressed by Eq. (3).

3 $$\Omega =2\pi *{f}_{1}=2\pi *\frac{RPM}{60}$$

The key to determining the number of blades lies in the harmonic structure of the signal. The relative intensity of the harmonics was found to be a direct function of the number of blades on the propeller. Specifically, for a propeller with n blades, the n^th^ harmonics exhibits strong intensity in both the simulation and experimental data. Additionally, it was observed, at least for 2, 3 and 4-blade drones, that an odd number of blades leads to stronger odd harmonics while an even number of blades tends to magnify even harmonics. Importantly, for drones with 4 blades, an additional spectral feature was observed: a "half fundamental" frequency component at half the fundamental frequency (500 Hz in the simulation). This component generates its own harmonics, which appear between the true harmonics of the signal (e.g., at 1500 Hz, 2500 Hz, etc.), providing an additional signature to identify 4-blade drones. The underlying principle of the presented method is that rotating propeller blades modulate the intensity of backscattered light in a periodic manner, generating a temporal signal whose frequency content reflects the blade properties. This is conceptually similar to micro-Doppler radar analysis, where motion-induced frequency shift modulations encode the dynamics of rotating or vibrating components [25, 26]. In the optical domain, this modulation manifests as amplitude variation due to changes in the apparent cross-section of the propeller. By performing Fourier analysis on the backscattered signal, we extract a spectral signature composed of a fundamental frequency (associated with the rotation rate) and its harmonics (related to blade number).

Thus, the frequency analysis of the backscattered signals provides both the fundamental frequency ${f}_{1}$ (and thus the RPM or angular velocity) and the number of blades N_b_ on the propeller. These two key parameters can be robustly extracted from the data, enabling a first step towards the differentiation between drone models. A notable advantage of the proposed methodology is that it does not require absolute calibration of the detected signal intensity. The frequency-domain analysis is based entirely on the temporal modulation of the backscattered signal caused by the rotating blades. As long as the detector exhibits a linear response with respect to changes in optical cross-section, the extraction of the number of blades remains valid regardless of the absolute signal level.

The agreement between the numerical simulations and experimental measurements is excellent. Both the time-domain signals and their corresponding frequency analyses exhibit similar features. As anticipated, the retrieved fundamental frequencies from the simulations match the expected value of 1000 Hz for all three simulated drones. In the experimental data, the fundamental frequencies are 660 Hz for the 2-blade drone, 876 Hz for the 3-blade drone, and 975 Hz for the 4-blade drone.

**Fig. 4:**
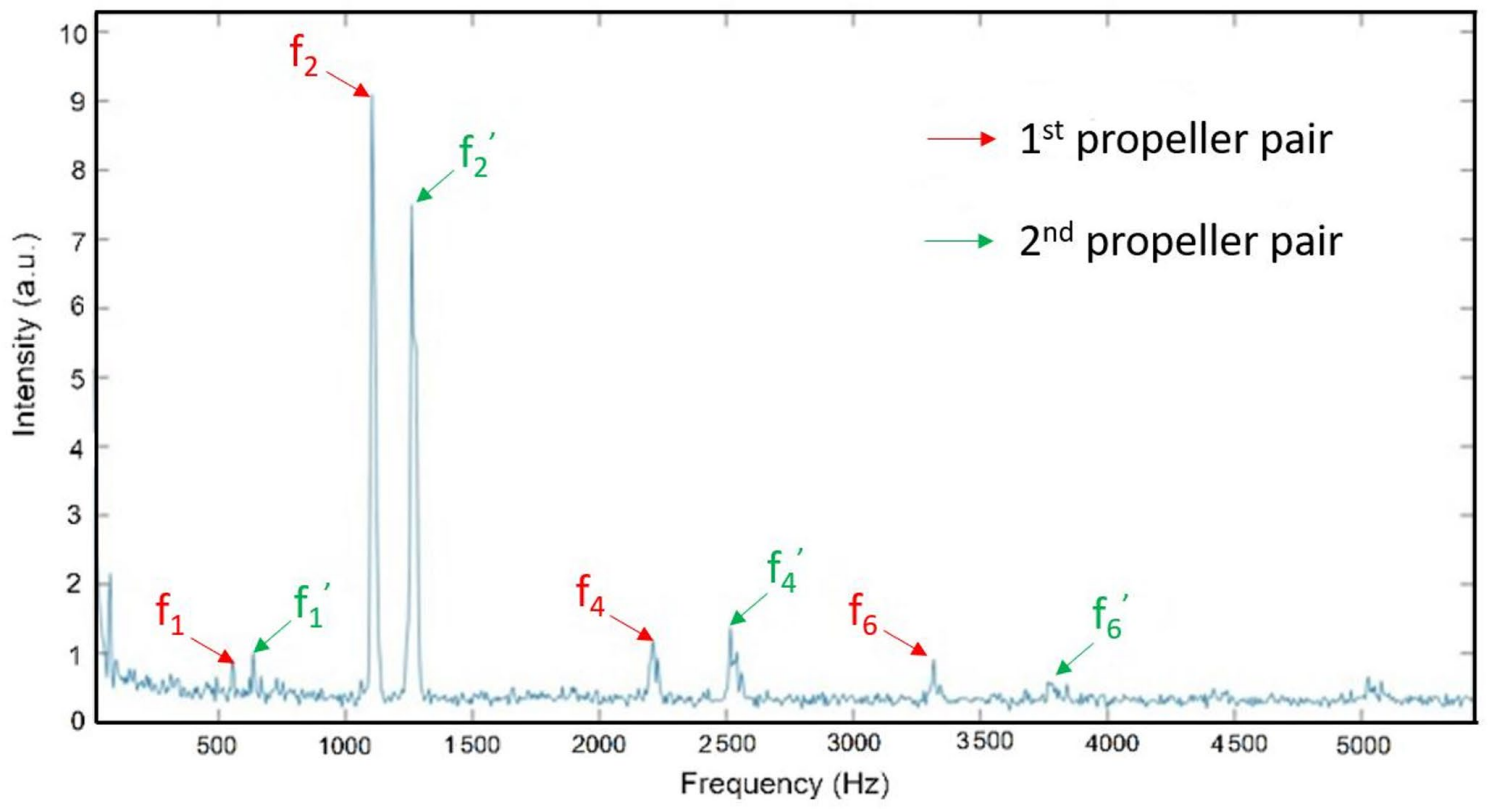
Fourier transform analysis of backscattered signal as a drone passes through the laser beam. From this experimental data, one may determine the number of blades, as well as the differential rotation rate between pairs of propellors indicating that the drone is maneuvering

Fourier analysis of the backscattered signal could potentially be leveraged to determine the number of propellers (and not just the blade number) on the drone by identifying closely spaced frequency peaks. These peaks arise because individual propellers often rotate at slightly different RPMs in order for the drone to navigate. The frequency separation between these peaks may provide insight into the different rotations of the propellers, which may also indicate changes in the drone's flight trajectory. For instance, Fig. 4 shows a prominent second harmonic peak (f_2_ and f_2_’) and no half-harmonics, correctly indicating that the drone is equipped with 2-blade propellers. In this example, two distinct sets of fundamental frequencies (f_1_ and f_1_’) and their corresponding harmonics can be distinguished. This suggests a difference in RPM between the front and rear (or left and right) propellers as the drone was adjusting its orientation by desynchronizing the rotation of its propellers. This rotation difference can serve as a valuable indicator of active or impending trajectory corrections, and could potentially be used as a predictive tool for tracking the drone's movement and flight behavior.

A limitation of the proposed methodology arises in scenarios where multiple UAVs simultaneously occupy the laser beam. In such cases, the backscattered signals from different targets are superimposed, complicating the frequency-domain analysis. If the individual drones exhibit sufficiently distinct propeller rotation speeds, their respective fundamental frequencies and harmonics may still be resolved, allowing partial separation of the signals. However, in cases of similar RPMs or overlapping harmonic structures, signal disentanglement becomes challenging, potentially limiting the reliability of the extracted features. That said, such overlapping is not expected to occur frequently under typical operating conditions, except in situations involving dense formations or swarms of UAVs. The robustness of the method may be influenced by external factors such as drone speed, distance, orientation, and payload, which affect signal duration, modulation contrast, and interpretability. A systematic study of these effects lies beyond the scope of the present work; however, their qualitative influence is discussed here to clarify practical limitations and applicability.

One consideration that may come to mind is the drone speed, however, the frequency analysis is not impacted by the drone movement, as long as the targeted UAV is within the laser beam, the backscattered signal will contain the necessary frequency components for the proposed methodology to work, regardless of the drone velocity. However, the ability to resolve the fundamental frequency and its harmonics requires to record a signal of sufficient temporal duration. In practice, very fast drones may be challenging: if the drone transits too quickly through the laser beam, the number of recorded oscillation cycles may be insufficient for reliable frequency-domain analysis. At a minimum, the drone must remain within the beam for at least two full propeller rotations. This constraint imposes efficient tracking of the UAV by the lidar system.

Another factor is the drone’s altitude, or more generally, its distance from the detection system. As distance increases, the backscattered signal amplitude decreases due to the 1/d^2^ dependence and atmospheric attenuation. This reduction in signal strength lowers the signal-to-noise ratio and can obscure higher-order harmonics, making blade count retrieval more difficult. While the method remains theoretically valid, the reliability of the extracted features may degrade with increasing altitude.

Technically the backscattered signal depends on the orientation of the drone in respect to the laser beam because the apparent cross-section of the propeller blades changes with viewing angle, yet the core principle of the analysis remains applicable. As long as there is a periodic variation in the apparent cross-section that is directly correlated to blade rotation, the method remains valid. This condition is satisfied across most observation angles in the simulation. However, since the simulation only include the rotating blades, it is observed that as the viewing angle approaches a direct top-down or bottom-up configuration (i.e., aligned with the rotation axis of the propellers) the modulation contrast tends toward zero in the mathematical limit. This outcome is expected due to the rotational symmetry of the propeller geometry, which leads to a constant apparent cross-section over time at those specific observation angles. In real UAVs, however, structural components such as the arms, central body or motor mounts intermittently block parts of the rotating blades or are themselves periodically obscured by the blades. For instance, when viewed from below, the blades may periodically pass behind the arms, while from above they may periodically pass in front of them, both cases introducing a time-dependent variation in the observed cross-section. These recurring obstructions modulate the total backscattered signal and preserve the periodic structure required for the frequency analysis. As a result, although the signal contrast and complexity may vary with viewing angle, the frequency-domain analysis remains applicable in practice across all realistic flight geometries.

Finally, another consideration is the drone's payload. In principle, if the number of propellers, the number of blades, their RPM, and the geometrical cross-section of the blades were known, it would be possible to estimate the lift and thus infer the payload. However, recovering the actual blade cross-section from the optical signal would require knowledge of the exact drone orientation, which is not available a priori. As a result, such analysis introduces additional complexity and assumptions and is not addressed in the present study. Nevertheless, this represents a promising direction for future investigations.

## Conclusion

In this study, we demonstrated the potential of a new frequency-domain analysis method for the characterization of drones by analyzing the backscattered optical signals produced by their rotating propellers.

Through a combination of numerical simulations and experimental measurements, we were able to show that some key physical characteristics of drones can be derived from optical signals. The frequency analysis of the backscattered signals allowed us to retrieve the fundamental frequency of the propellers, directly related to their rotation speed, as well as to identify the number of blades per propeller based on the harmonic structure of the signal.

Although the present experimental setup employs a 940 nm continuous-wave laser at relatively high power to ensure strong signal quality under laboratory conditions, we note that the methodology is fully compatible with eye-safe and field-ready architectures. In particular, lasers operating at 1550 nm, a mature and widely used wavelength band for remote sensing, offer advantages in terms of human eye safety, system miniaturization, and fiber-based integration. With appropriate detection strategies, these systems could achieve comparable or enhanced performance at significantly lower power levels, making them well suited for portable and deployable UAV monitoring applications.

This methodology could serve as a valuable tool for tracking drones during maneuvering, adding an additional layer of insight to UAV monitoring systems. Combined with additional sensors, tracking the propeller’s RPM could lead to the detection of potential payload as the additional weight would require faster RPM from the propellers. The ability to retrieve the number of blades can help in the identification of the drone model while the detection of closely spaced frequency components could provide information about the differential rotation of multiple propellers, potentially indicating changes in the drone’s flight trajectory.

While the focus of this paper has been on the extraction of RPM and blade count, the presented methodology opens the door to several exciting future research avenues in the use of lidar for drone characterization.

## Data Availability

The data presented in this study are available on request from the corresponding author.
